# Machine Learning Tools for Image-Based Glioma Grading and the Quality of Their Reporting: Challenges and Opportunities

**DOI:** 10.3390/cancers14112623

**Published:** 2022-05-25

**Authors:** Sara Merkaj, Ryan C. Bahar, Tal Zeevi, MingDe Lin, Ichiro Ikuta, Khaled Bousabarah, Gabriel I. Cassinelli Petersen, Lawrence Staib, Seyedmehdi Payabvash, John T. Mongan, Soonmee Cha, Mariam S. Aboian

**Affiliations:** 1 Department of Radiology and Biomedical Imaging, Yale School of Medicine, 333 Cedar Street, P.O. Box 208042, New Haven, CT 06520, USA; sara.merkaj@uni-ulm.de (S.M.); ryan.bahar@yale.edu (R.C.B.); tal.zeevi@yale.edu (T.Z.); mingde.lin@yale.edu (M.L.); ichiro.ikuta@yale.edu (I.I.); gabriel.cassinellipetersen@yale.edu (G.I.C.P.); lawrence.staib@yale.edu (L.S.); sam.payabvash@yale.edu (S.P.); 2 Department of Neurosurgery, University of Ulm, Albert-Einstein-Allee 23, 89081 Ulm, Germany; 3 Visage Imaging, Inc., 12625 High Bluff Dr, Suite 205, San Diego, CA 92130, USA; 4 Visage Imaging, GmbH., Lepsiusstraße 70, 12163 Berlin, Germany; kbousabarah@visageimaging.com; 5 Department of Radiology and Biomedical Imaging, University of California San Francisco, 505 Parnassus Ave., San Francisco, CA 94143, USA; john.mongan@ucsf.edu (J.T.M.); soonmee.cha@ucsf.edu (S.C.)

**Keywords:** artificial intelligence, glioma, machine learning, deep learning, reporting quality

## Abstract

**Simple Summary:**

Despite their prevalence in research, ML tools that can predict glioma grade from medical images have yet to be incorporated clinically. The reporting quality of ML glioma grade prediction studies is below 50% according to TRIPOD—limiting model reproducibility and, thus, clinical translation—however, current efforts to create ML-specific reporting guidelines and risk of bias tools may help address this. Several additional deficiencies in the areas of ML model data and glioma classification hamper widespread clinical use, but promising efforts to overcome current challenges and encourage implementation are on the horizon.

**Abstract:**

Technological innovation has enabled the development of machine learning (ML) tools that aim to improve the practice of radiologists. In the last decade, ML applications to neuro-oncology have expanded significantly, with the pre-operative prediction of glioma grade using medical imaging as a specific area of interest. We introduce the subject of ML models for glioma grade prediction by remarking upon the models reported in the literature as well as by describing their characteristic developmental workflow and widely used classifier algorithms. The challenges facing these models—including data sources, external validation, and glioma grade classification methods —are highlighted. We also discuss the quality of how these models are reported, explore the present and future of reporting guidelines and risk of bias tools, and provide suggestions for the reporting of prospective works. Finally, this review offers insights into next steps that the field of ML glioma grade prediction can take to facilitate clinical implementation.

## 1. Introduction

### 1.1. Artificial Intelligence, Machine Learning, and Radiomics

Innovations in computation and imaging have rapidly enhanced the potential for artificial intelligence (AI) to impact diagnostic neuroradiology. Emerging areas of implementation include AI in stroke (e.g., early diagnosis, detection of large vessel occlusion, and outcome prediction) [1], AI in spine (fracture detection, and vertebrae segmentation) and detection of intracranial aneurysms and hemorrhage [2], among other disciplines. Machine learning (ML) and its subfield, deep learning (DL), are branches of AI that have received particular attention. ML algorithms, including DL, decipher patterns in input data and independently learn to make predictions [3]. The advent of radiomics—which mines data from images by transforming them into features quantifying tumor phenotypes—has fueled the application of ML methods to imaging, including radiomics-based ML analysis of brain tumors [4,5,6]. Commonly extracted radiomic features include shape and size, texture, first-order, second-order, higher-order features, etc. (Table 1).

### 1.2. Machine Learning Applications in Neuro-Oncology

As the most common primary brain tumors, gliomas constitute a major focus of ML applications to neuro-oncology [7,8]. Prominent domains of glioma ML research include the image-based classification of tumor grade and prediction of molecular and genetic characteristics. Genetic information is not only instrumental to tumor diagnosis in the 2021 World Health Organization classification, but also significantly affects survival and underpins sensitivity to therapeutic interventions [9,10]. ML-based models for predicting tumor genotype can therefore guide earlier diagnosis, estimation of prognosis, and treatment-related decision-making [11,12]. Other significant areas of glioma ML research relevant to neuroradiologists include automated tumor segmentation on MRI, detection and prediction of tumor progression, differentiation of pseudo-progression from true progression, glioma survival prediction and treatment response, distinction of gliomas from other tumors and non-neoplastic lesions, heterogeneity assessment based on imaging features, and clinical incorporation of volumetrics [13,14,15]. Furthermore, ML tools may optimize neuroradiology workflow by expediting the time to read studies from image review to report generation [16]. As an image interpretation support tool, ML importantly may improve diagnostic performance [17,18]. Prior works demonstrate that AI alone can approach the diagnostic accuracy of neuroradiologists and other sub-specialty radiologists [19,20,21].

### 1.3. Image-Based Machine Learning Models for Glioma Grading

This review is concerned with the growing body of studies developing predictive ML models for image-based glioma grading, a fundamentally heterogeneous area of literature. While numerous ML models exist to predict high-grade gliomas and low-grade gliomas, they vary in their definitions of high- and low-grade [22,23,24]. Other models predict individual glioma grades (e.g., 2 vs. 3, 3 vs. 4), but few have combined glioma grading with molecular classification despite the incorporation of both grade and molecular subtype in 2016 World Health Organization central nervous system tumor classification [25,26]. While studies focus on MRI, they are diverse in the sequences used for prediction, with earlier publications relying on conventional imaging and increasing incorporation of advanced MRI sequences throughout the years [27,28,29,30]. Finally, studies vary considerably in their feature extraction and selection methods, datasets, validation techniques, and classification algorithms [31].

It is our belief that the ML models with potential to support one of the most fundamental tasks of the neuroradiologist—glioma diagnosis—present obstacles and opportunities relevant to the radiology community, especially as radiologists endeavor to bring ML models into clinical practice. In this article, we aim to introduce the subject of developing ML models for glioma grade prediction, highlight challenges facing these models and their reporting within the literature, and offer insights into next steps the field can take to facilitate clinical implementation.

## 2. Workflow for Developing Prediction Models

Despite their heterogeneity, ML glioma grade prediction studies follow similar steps in developing their models. The development workflow starts with acquisition, registration, and pre-processing (if necessary) of multi-modal MR images. Common pre-processing tasks include data cleaning, normalization, transformation, and dealing with incomplete data, among other tasks [32]. An in-depth exploration of pre-processing is beyond the scope of this review and readers should refer to Kotsiantis et al. for further explanation. Next, tumors undergo segmentation—the delineation of tumor, necrosis, and edema borders—which can be a manual, semi-automatic, or fully automatic process. Manual segmentations rely on an expert delineating and annotating Regions of Interest (ROIs) by hand. Semi-automated segmentations generate automated ROIs that need to be checked and modified by experts. Fully automatic segmentations, on the other hand, are DL-generated (most frequently by convolutional neural networks (CNNs)), which automatically delineate ROIs and omit the need for manual labor [33]. In general, semi-automated segmentations are considered to be more reliable and transparent than fully automatic segmentations. However, they are less time-efficient than automatic segmentations and always require manual input from experts in the field. Whereas manual segmentation is laborious, time-consuming, and subject to inter-reader variability, fully automatic deep-learning generated segmentations may potentially overcome these challenges [34].

Feature extraction is then performed to extract qualitative and quantitative information from imaging. Commonly extracted data include radiomic features (shape, first-order, second-order, higher-order features, etc.), clinical features (age, sex, etc.), and tumor-specific Visually AcceSAble Rembrandt Images (VASARI) features. Feature types and their explanations are presented in Table 1.

Open-source packages such as PyRadiomics have been developed as a reference standard for radiomic feature extraction [36]. Clinical features are known to be important markers for predicting glioma grades and molecular subtypes [37]. VASARI features, developed by The Cancer Imaging Archive (TCIA), are frequently found in studies that qualitatively describe tumor morphology using visual features and controlled vocabulary/standardized semantics [38].

Current technology permits extraction of over 1000 features per image. As a high number of features may lead to model overfitting, model developers commonly reduce the number of features used through feature selection. Feature selection methods, including Filter, Wrapper, and Embedded methods, remove non-informative features that reduce the model’s overall performance [39].

**Figure 1:**
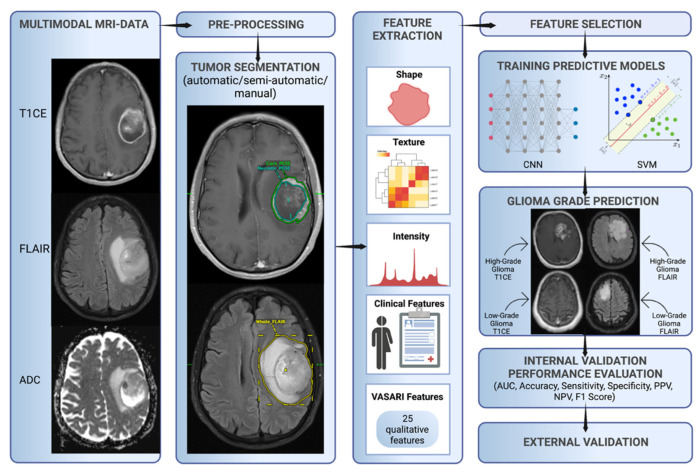
Characteristic workflow for developing ML glioma grade prediction models. VASARI = Visually AcceSAble Rembrandt Images, AUC = area under the curve receiver operating characteristic, CNN = convolutional neural network, ML = machine learning, NPV = negative predictive value, PPV = positive predictive value, and SVM = support vector machine.

The final set of features is fed into a glioma grade classification algorithm(s)—for example, support vector machine (SVM) and CNN—during the training process. The classifier performance is then measured through performance metrics such as accuracy, area under the curve receiver operating characteristic, sensitivity, specificity, positive predictive value, negative predictive value, and F1 score. The model is validated internally, usually through hold-out or cross-validation techniques. Ideally, the model is externally validated as a final step to ensure reproducibility, generalizability, and reliability in a different setting (Figure 1).

## 3. Algorithms for Glioma Grade Classification

The most common high-performing ML classifiers for glioma grading in the literature are SVM and CNN [13]. SVM is a classical ML algorithm that represents objects as points in an n-dimensional space, with features serving as coordinates. SVMs use a hyperplane, or an n-1 dimensional subspace, to divide the space into disconnected areas [40]. These distinct areas represent the different classes that the model can classify. Unlike CNNs, SVMs require hand-engineered features, such as from radiomics, to serve as inputs. This requirement may be advantageous for veteran diagnostic imagers, whose knowledge of brain tumor appearance may enhance feature design and selection. Hand-engineered features also can undergo feature reduction to mitigate the risks of overfitting, and prior works demonstrate better performance for glioma grading models using a smaller number of quantitative features [41]. However, hand-engineered features are limited since they cannot be adjusted during model training, and it is uncertain if they are optimal features for classification. Moreover, hand-engineered features may not generalize well beyond the training set and should be tested extensively prior to usage [42,43].

CNNs are a form of deep learning based on image convolution. Images are the direct inputs to the neural network, rather than the manually engineered features of classical ML. Numerous interconnected layers each compute feature representations and pass them on to subsequent layers [43,44]. Near the network output, features are flattened into a vector that performs the classification task. CNNs appeared for glioma grading in 2018 and have risen quickly in prevalence while exhibiting excellent predictive accuracies [45,46,47,48]. To a greater extent than classical ML, they are suited for working with large amounts of data, and their architecture can be modified to optimize efficiency and performance [46]. Disadvantages include the opaque “black box” nature of deep learning and associated difficulty with interpreting model parameters, along with problems that variably apply to classical ML as well (e.g., high amount of time and data required for training, hardware costs, and necessary user expertise) [49,50].

In our systematic review of 85 published ML studies developing models for image-based glioma grading, we found SVM and CNN to have mean accuracies of 90% and 91%, respectively [51]. Mean accuracies for these algorithms were similar across classification tasks regardless of whether the classification was binary or multi-class (e.g., 90% for the 24 studies whose best models performed binary classification of grades 1/2 vs. 3/4 compared to 86% for the 5 studies classifying grade 2 vs. 3 vs. 4). No consensus has been reached regarding the optimal ML algorithm for image-based glioma classification.

## 4. Challenges in Image-Based ML Glioma Grading

### 4.1. Data Sources

Since 2011, a significant number of ML glioma grade prediction studies have used open-source multi-center datasets to develop their models. BraTS [52] and TCIA [53] are two prominent public datasets that contain multi-modal MRI images of high- and low-grade gliomas and patient demographics. BraTS was first made available in 2012, with the 2021 dataset containing 8000 multi-institutional, multi-parametric MR images of gliomas [52]. TCIA first went online in 2011 and contains MR images of gliomas collected across 28 institutions [53]. These datasets were developed with the aim of providing a unified multi-center resource for glioma research. A variety of predictive models have been trained and tested on these large datasets since their 2011 release [54]. Despite their value as public datasets for model development, several limitations should be considered. Images are collected across multiple institutions with variable protocols and image quality. Co-registration and imaging pre-processing integrate these images into a single system. Although these techniques are necessary, they may reduce heterogeneity within the datasets [52]. Models developed on these datasets may perform well in training and testing. Nevertheless, the results may not be reproducible in the real-world clinical setting, where images and tumor presentations are heterogeneous. We strongly support large multi-center datasets in order to demonstrate model performance across distinct hospital settings. We, however, recommend such initiatives incorporate images of various diagnostic qualities into their training datasets, which more closely resemble what is seen in daily practice.

### 4.2. External Validation

Publications have reported predictive models for glioma grading throughout the last 20 years with the majority relying on internal validation techniques, of which cross-validation is the most popular. While internal validation is a well-established method for measuring how well a model will perform on new cases from the initial dataset, additional evaluation on a separate dataset (i.e., external validation) is critical to demonstrate model generalizability. External validation mitigates site bias (differences amongst centers in protocols, techniques, scanner variability, level of experience, etc.) and sampling/selection bias (performance only applicable to the specific training set population/demographics) [55]. Not controlling for these two major biases undermines model generalizability, yet few publications externally validate their models [13]. Therefore, normalizing external validation is a crucial step in developing glioma grade prediction models that are suitable for clinical implementation.

### 4.3. Glioma Grade Classification Systems

The classification of glioma subtypes into high- and low-grade gliomas is continuously evolving. In 2016, an integrated histological–molecular classification replaced the previous purely histopathological classification [56]. In 2021, the Consortium to Inform Molecular and Practical Approaches to CNS Tumor Taxonomy (cIMPACT NOW) once more accentuated the diagnostic value of molecular markers, such as the isocitrate dehydrogenase mutation, for glioma classification [57]. As a result of the evolving glioma classification system, definitions for high- and low-grade gliomas vary across ML glioma grade prediction studies and publication years. This reduces the comparability of models themselves and grade-labeled datasets used for model development. We recommend future glioma grade prediction studies focus on both glioma grade and molecular subtypes for more comprehensive and reliable results over time. Neuropathologic diagnostic emphasis has shifted from purely based on microscopic histology to one that combines morphologic and molecular genetic features of tumor including gene mutations, chromosomal copy number alterations, and gene rearrangements to yield integrated diagnosis. Rapid developments in next generation sequencing techniques, multimodal molecular analysis, large scale genomic and epigenomic analyses, and DNA methylation methods promise to fundamentally transform the pathologic CNS tumor diagnostics including glioma diagnosis and grading to whole another level of precision and complexity.

**Figure 2:**
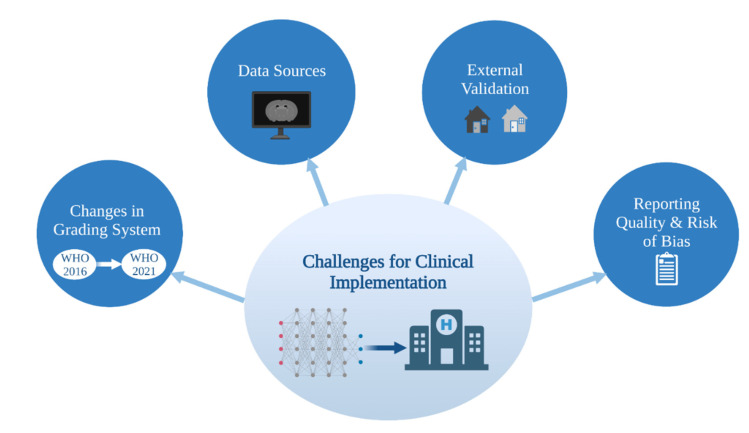
Challenges for clinical implementation of ML glioma grade prediction models. ML = machine learning. WHO = World Health Organization.

Current and future ML methods must keep abreast of the rapid progress in tissue based integrated diagnostics in order to contribute to and make an impact on the clinical care of glioma patients (Figure 2).

### 4.4. Reporting Quality and Risk of Bias

#### 4.4.1. Overview of Current Guidelines and Tools for Assessment

It is critical that studies detailing prediction models, such as those for glioma grading, exhibit a high caliber of scientific reporting in accordance with consensus standards. Clear and thorough reporting enables more complete understanding by the reader and unambiguous assessment of study generalizability, quality, and reproducibility, encouraging future researchers to replicate and use models in clinical contexts. Several instruments have been designed to improve the reporting quality (defined here as the transparency and thoroughness with which authors share key details of their study to enable proper interpretation and evaluation) of studies developing models. The Transparent Reporting of a multivariable prediction model for Individual Prognosis or Diagnosis (TRIPOD) Statement was created in 2015 as a set of recommendations for studies developing, validating, or updating diagnostic or prognostic models [58]. The TRIPOD Statement is a checklist of 22 items considered essential for transparent reporting of a prediction model study. In 2017, with a concurrent rise in radiomics-based model studies, the radiomics quality score (RQS) emerged [59]. RQS is an adaptation of the TRIPOD approach geared toward a radiomics-specific context. The tool has been used throughout the literature for evaluating the methodological quality of radiomics studies, including applications to medical imaging [60]. Radiomics-based approaches for interpreting medical images have evolved to encompass the AI techniques of classical ML and, most recently, deep learning models. Most recently, in recognition of the growing need for an evaluation tool specific to AI applications in medical imaging, the Checklist for AI in Medical Imaging (CLAIM) was published in 2020 [61]. The 42 elements of CLAIM aim to be a best practice guide for authors presenting their research on applications of AI in medical imaging, ranging from classification and image reconstruction to text analysis and workflow optimization. Other tools—the Quality Assessment of Diagnostic Accuracy Studies (QUADAS-2) tool [62] and Prediction model Risk Of Bias ASsessment Tool (PROBAST) [63]—importantly evaluate the risk of bias in studies based on what is reported about their models (Table 2). Bias relates to systematic limitations or flaws in study design, methods, execution, or analysis that distort estimates of model performance [62]. High risk of bias discourages adaptation of the reported model outside of its original research context, and, at a systemic level, undermines model reproducibility and translation into clinical practice.

#### 4.4.2. Reporting Quality and Risk of Bias in Image-Based Glioma Grade Prediction

Assessments of ML-based prediction model studies have demonstrated that risk of bias is high and reporting quality is inadequate. In their systematic review of prediction models developed using supervised ML techniques, Navarro et al. found that the high risk of study bias, as assessed using PROBAST, stems from small study size, poor handling of missing data, and failure to deal with model overfitting [64]. Similar findings have been reported for glioma grade prediction literature. In our prior study conducting a TRIPOD analysis of more than 80 such model development studies, we report a mean adherence rate to TRIPOD of 44%, indicating poor quality of reporting [51]. Areas for improvement included reporting of titles and abstracts, justification of sample size, full model specification and performance, and participant demographics, and missing data. Sohn et al.’s meta-analysis of radiomics studies differentiating high- and low-grade gliomas estimated a high risk of bias according to QUADAS-2, attributing this to the fact that all their analyzed studies were retrospective (and have the potential for bias because patient outcomes are already known), the lack of control over acquisition factors in the studies using public imaging data, and unclear study flow and timing due to poor reporting [41]. Readers should refer directly to Navarro et al., Bahar et al. and Sohn et al. for more detailed discussion of shortcomings in study reporting and risk of bias.

#### 4.4.3. Future of Reporting Guidelines and Risk of Bias Tools for ML Studies

Efforts by authors to refine how they report their studies depend upon existing reporting guidelines. In their systematic review, Yao et al. identified substantial limitations to neuroradiology deep learning reporting standardization and reproducibility [65]. They recommended that future researchers propose a reporting framework specific to deep learning studies. This call for an AI-targeted framework parallels contemporary movements to produce AI extensions of established reporting guidelines. TRIPOD creators have discussed the challenges with ML not captured in the TRIPOD Statement [66]. The introduction of more relevant terminology and movement away from regression-based model approaches will be a part of the forthcoming extension of TRIPOD for studies reporting ML-based diagnostic or prognostic models (TRIPOD-AI) [66,67]. QUADAS-2 creators also announced a plan for an AI-extension (QUADAS-AI), noting that their tool similarly does not accommodate AI-specific terminology and further documenting sources of AI study bias that are not signaled by the tool [68]. PROBAST-AI is in development too [66].

#### 4.4.4. Recommendations

Systematic reviews and meta-analyses in the field [41,51,64] reveal various aspects of reporting and bias risk that need to be addressed in order to promote complete understanding, rigorous assessment, and reproducibility of image-based ML glioma grading studies. Based on the problems identified in this literature (discussed in 4.4.2), we encourage future works to closely adhere to the reporting and risk of bias tools and guidelines most relevant to them, with particular attention to:

- Clearly signifying the development of a prediction model in their titles;
- Increasing the number of participants included in training/testing/validation sets;
- Justifying their choice of sample/sample size (whether that be on practical or logistical grounds) and approach to handling missing data (e.g., imputation);
- Specifying all components of model development (including data pre-processing and model calibration) and a full slate of performance metrics (accuracy, area under the receiver operating characteristic curve (AUC), sensitivity, specificity, positive predictive value, negative predictive value, and F1 score as well as associated confidence intervals) for training/testing/validation. While accuracy is the most comprehensive measure of model performance, AUC is more sensitive to performance differences between classes (e.g., within imbalanced datasets) and should always be reported [69];
- Providing open access to the source code of their algorithms.

For prediction model studies that involve applications of AI to medical imaging, CLAIM is the only framework that is specific to AI and able to capture the nuances of their model reporting—including data preprocessing steps, model layers/connections, software libraries and packages, initialization of model parameters, performance metrics of models on all data partitions, and public access to full study protocols. We, therefore, recommend future studies developing ML models for the prediction of glioma grade from imaging use CLAIM to guide how they present their work. The authors should remain vigilant regarding the release of other AI-specific frameworks that may best suit their studies and seek out AI-specific risk of bias tools to supplement CLAIM once available.

## 5. Future Directions

**Figure 3:**
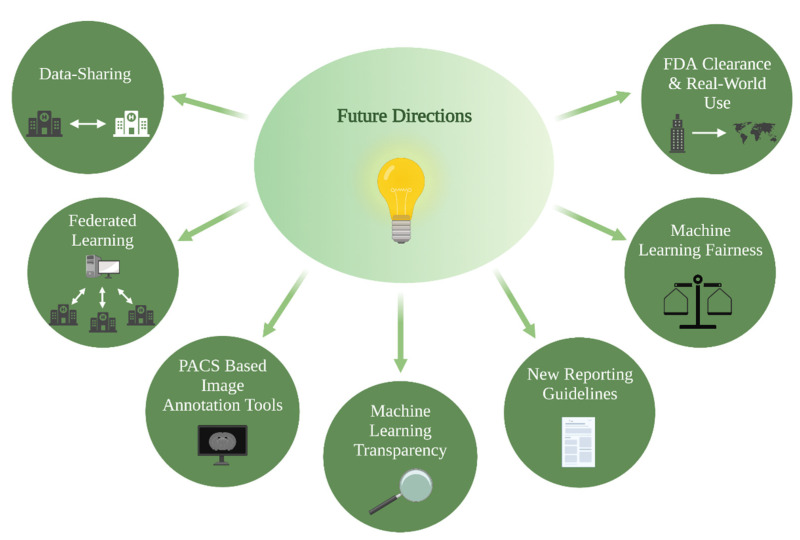
Future directions for clinical implementation of ML glioma grade prediction models, ML = machine learning.

ML models present an attractive solution towards overcoming current barriers and accelerating the transition to patient-tailored treatments and precision medicine. Novel algorithms combine information derived from multimodal imaging to molecular markers and clinical information, with the aim of bringing personalized predictions on a patient level into routine clinical care. Relatedly, multi-omic approaches that integrate a variety of advanced techniques such as proteomics, transcriptomics, epigenomics, etc., are increasingly gaining importance in understanding cancer biology and will play a key role in the facilitation of precision medicine [70,71]. The growing presence of ML models in research settings is indisputable, yet several strategies should be considered to facilitate clinical implementation: PACS-based image annotation tools, data-sharing and federated learning, ML fairness, ML transparency, and FDA clearance and real-world use (Figure 3).

### 5.1. PACS-Based Image Annotation Tools

Large, annotated datasets that are tailored to the patient populations of individual hospitals and practices are key to training clinically applicable prediction algorithms. An end-to-end solution for generation of these datasets, in which all steps of the ML workflow are performed automatically in clinical picture archiving and communication system (PACS) as the neuroradiologist reads a study, is considered the “holy grail” of AI workflow in radiology [72]. A mechanism for achieving this is through automated/semi-automated segmentation, feature extraction, and prediction algorithms embedded into clinical PACS that provide reports in real-time. The accumulation of saved segmentations through this workflow could accelerate the generation of large, annotated datasets, in addition to providing a decision-support tool for neuroradiologists in daily practice. Under these circumstances, establishing strong academic-industry partnerships for the development of clinically useful image annotation tools is fundamental.

### 5.2. Data-Sharing and Federated Learning

Multi-institutional academic partnerships are also critical for maximizing clinical applications of ML. Data-sharing efforts are under way in order to accelerate the pace of research [73]. Cross-institutional collaborations not only enrich the quality of the input that goes into training the model, but also provide datasets for externally validating other institutions’ models. However, data-sharing across institutions is often hindered by technical, regulatory, and privacy concerns [74]. A promising solution for this is federated learning, an up-and-coming collaborative algorithm training effort that does not require cross-institutional data-sharing. In federated learning, models are trained locally inside an institution’s firewalls and learned weights or gradients are transferred from participating institutions for aggregation into a more robust model [75]. This overcomes the barriers of data-sharing and has been shown to be superior to algorithms trained on single-center datasets [76]. Federated learning is not without drawbacks, however; it depends on existing standards for data quality, protocols, and heterogeneity of data distribution. Researchers do not have access to model training data and may face difficulty interpreting unexpected results.

### 5.3. ML Fairness

A common misconception about AI algorithms is that they are not vulnerable to biases during decision-making. In reality, algorithm unfairness—defined as prejudice or discrimination that skews decisions toward individuals or groups based on their characteristics—has been extensively documented across AI applications. A well-known example is the Correctional Offender Management Profiling for Alternative Sanctions score, which was a tool that assisted judges with their decision to release an offender or keep them in prison. The software was found to be biased towards African Americans, judging them to be at higher risk for recommitting crimes compared to Caucasian individuals [77]. Additional examples of bias have been demonstrated across widely deployed biobanks [78], clinical trial accrual populations [79] and ICU mortality and 30-day psychiatric readmission prediction algorithms [80] among other medical domains. Publicly available tools, including Fairlearn and AI Fairness 360, assess and correct for algorithm unfairness ranging from allocation harms and quality of service harms to feature and racial bias [81,82]. These tools have yet to be applied widely in medical contexts despite their promising utility. Future works on AI in neuro-oncology should consider implementing evidence-based bias detection and mitigation tools tailored to their algorithm development setting and target population prior to clinical integration.

### 5.4. ML Transparency

The opaqueness of ML models—DL in particular—poses a barrier to their acceptance and usage. In addition, traditional measures such as software validation are insufficient for fulfilling legal, compliance, and/or other requirements for ML tool clarification [83,84]. Explainable artificial intelligence (xAI) approaches may address these concerns by explaining particular prediction outputs and overall model behavior in human-understandable terms [85]. A recent study demonstrates the successful use of state-of-the-art xAI libraries incorporating visual analytics for glioma classification [83]. Other approaches such as Grad-CAM generate visual explanations of DL model decisions and, therefore, enhance algorithm transparency [86]. These tools can support the interpretability of ML model outputs for future research as well as prime ML for dissemination and acceptance in clinical neuroradiology. Guidelines for authors, along with reporting quality assessment and risk of bias tools, should consider encouraging such approaches to further the transparency of literature in the field.

Of relevance to ML model transparency are the concepts of usability and causability. Usability can be defined as the ease of use of a computer system for users, or in other words, the extent to which a user and a system may communicate through an interface without misunderstanding [87,88]. Highly usable tools are associated with positive user satisfaction and performance in the field of human–computer interaction [89]. Causability is a parallel concept to usability and foundational for human–AI interaction. Causability reflects the understandability of an AI model (e.g., CNN) to a human as communicated by an explanation interface [89]. Causability, furthermore, determines relative importance and justifies what should be explained and how [90]. Embracing causability in the development of human–AI interfaces will help people understand the decision-making process of ML algorithms and improve trust. We believe this will lower the threshold for clinical ML utilization.

### 5.5. FDA Clearance and Real-World Use

Thousands of studies pertaining to applications of AI and ML in medical imaging have been published [15,82]. Yet, few imaging AI/ML algorithms have been cleared by the FDA as medical products [91], perhaps due in part to the lack of standardization and transparency in the FDA clearance process [92]. Bridging the gap between AI/ML research and FDA clearance—as well as FDA clearance and real-world algorithm use—will streamline the adoption of ML models for glioma grading into clinical settings. To this end, Lin presents several suggestions [93]. Partnering of the FDA with professional societies could facilitate the standardization of algorithm development and evaluation. A key focus would be resolving the split between how results are communicated in the literature (e.g., performance metrics) and what is relevant for AI product assessment (e.g., return on investment, integration and flexibility with PACS, ease of use, etc.). Moreover, reporting of post-marketing surveillance could help real-world use and algorithm performance drift.

## 6. Conclusions

ML glioma grade prediction tools are increasingly prevalent in research but have yet to be incorporated clinically. The reporting quality of ML glioma grade prediction studies is low, limiting model reproducibility and thus preventing reliable clinical translation. However, current efforts to create ML-specific reporting guidelines and risk of bias tools may help address these issues. Future directions for supporting clinical implementation of ML prediction models include data-sharing, federated learning, and development of PACS-based image annotation tools for the generation of large image databases, among other opportunities.

## Figures and Tables

**Table 1:** Overview of commonly extracted feature types in studies developing ML prediction models.

Feature Type	Explanation
Clinical	Describe patient demographics, e.g., gender and age.
Deep learning extracted	Derived from pre-trained deep neural networks.
First-order	Create a three-dimensional (3D) histogram out of tumor volume characteristics, from which mean, median, range, skewness, kurtosis, etc., can be calculated [35].
Higher-order	Identify repetitiveness in image patterns, suppress noise, or highlight details [35].
Qualitative	Describe visible tumor characteristics on imaging using controlled vocabulary, e.g., VASARI features (tumor location, side of lesion center, enhancement quality, etc.).
Second-order	Classify texture characteristics, e.g., contrast, correlation, dissimilarity, maximum probability, grey level run length features, etc. [35]
Shape and size	Describe the statistical inter-relationships between neighboring voxels, e.g., total volume or surface area, surface-to-volume ratio, tumor compactness, sphericity, etc. [35]

**Table 2:** Overview of major reporting guidelines and bias assessment tools for diagnostic and prognostic studies.

Guideline/Tool	Full Name	Year Published	Articles Targeted	Purpose	Specific to ML?
QUADAS-2 ^4^	Quality Assessment of Diagnostic Accuracy Studies	2011 (original QUADAS ^4^: 2003)	Diagnostic accuracy studies	Evaluates study risk of bias and applicability	No; QUADAS-AI ^4^ is in development
TRIPOD ^6^	Transparent Reporting of a multivariable prediction model for Individual Prognosis Or Diagnosis	2015	Studies developing, validating, or updating a diagnostic or prognostic prediction model	Provides a set of recommendations for study reporting	No; TRIPOD-AI ^6^ is in development
RQS ^5^	Radiomics quality score	2017	Radiomic studies	Assesses study quality (emulating TRIPOD ^6^)	No
PROBAST ^3^	Prediction model Risk Of Bias ASsessment Tool	2019	Studies developing, validating, or updating a diagnostic or prognostic prediction model	Evaluates study risk of bias and applicability	No; PROBAST-AI ^3^ is in development
CLAIM ^2^	Checklist for AI ^1^ in Medical Imaging	2020	AI ^1^ studies in medical imaging	Guides authors in presenting (and aids reviewers in evaluating) their research	Yes

^1^ AI = artificial intelligence, ^2^ CLAIM = Checklist for AI in Medical Imaging, ^3^ PROBAST = Prediction model Risk Of Bias ASsessment Tool, ^4^ QUADAS-2 = Quality Assessment of Diagnostic Accuracy Studies, ^5^ RQS = radiomics quality score, and ^6^ TRIPOD = Transparent Reporting of a multivariable prediction model for Individual Prognosis or Diagnosis.

## Data Availability

Not applicable.
